# Associations of incidental vertebral fractures and longitudinal changes of MR–based proton density fat fraction and T2* measurements of vertebral bone marrow

**DOI:** 10.3389/fendo.2022.1046547

**Published:** 2022-11-17

**Authors:** Yannik Leonhardt, Jannik Ketschau, Stefan Ruschke, Florian T. Gassert, Leander Glanz, Georg C. Feuerriegel, Felix G. Gassert, Thomas Baum, Jan S. Kirschke, Rickmer F. Braren, Benedikt J. Schwaiger, Marcus R. Makowski, Dimitrios C. Karampinos, Alexandra S. Gersing

**Affiliations:** ^1^ Department of Radiology, Klinikum Rechts der Isar, School of Medicine, Technical University of Munich, Munich, Germany; ^2^ Department on Neuroradiology, Klinikum Rechts der Isar, School of Medicine, Technical University of Munich, Munich, Germany; ^3^ Department of Neuroradiology, University Hospital of Munich (LMU), Munich, Germany

**Keywords:** osteoporosis, magnetic resonance imaging, bone density, PDFF, chemical shift encoding-based water-fat separation

## Abstract

**Background:**

Quantitative magnetic resonance imaging (MRI) techniques such as chemical shift encoding-based water-fat separation techniques (CSE-MRI) are increasingly applied as noninvasive biomarkers to assess the biochemical composition of vertebrae. This study aims to investigate the longitudinal change of proton density fat fraction (PDFF) and T2* derived from CSE-MRI of the thoracolumbar vertebral bone marrow in patients that develop incidental vertebral compression fractures (VCFs), and whether PDFF and T2* enable the prediction of an incidental VCF.

**Methods:**

In this study we included 48 patients with CT-derived bone mineral density (BMD) measurements at baseline. Patients that presented an incidental VCF at follow up (*N*=12, mean age 70.5 ± 7.4 years, 5 female) were compared to controls without incidental VCF at follow up (*N*=36, mean age 71.1 ± 8.6 years, 15 females). All patients underwent 3T MRI, containing a significant part of the thoracolumbar spine (Th11-L4), at baseline, 6-month and 12 month follow up, including a gradient echo sequence for chemical shift encoding-based water-fat separation, from which PDFF and T2* maps were obtained. Associations between changes in PDFF, T2* and BMD measurements over 12 months and the group (incidental VCF vs. no VCF) were assessed using multivariable regression models. Mixed-effect regression models were used to test if there is a difference in the rate of change in PDFF, T2* and BMD between patients with and without incidental VCF.

**Results:**

Prior to the occurrence of an incidental VCF, PDFF in vertebrae increased in the VCF group (Δ_PDFF_=6.3 ± 3.1%) and was significantly higher than the change of PDFF in the group without VCF (Δ_PDFF_=2.1 ± 2.5%, *P*=0.03). There was no significant change in T2* (Δ_T2*_=1.7 ± 1.1ms vs. Δ_T2*_=1.1 ± 1.3ms, *P*=0.31) and BMD (Δ_BMD_=-1.2 ± 11.3mg/cm^3^ vs. Δ_BMD_=-11.4 ± 24.1mg/cm^3^, *P*= 0.37) between the two groups over 12 months. At baseline, no significant differences were detected in the average PDFF, T2* and BMD of all measured vertebrae (Th11-L4) between the VCF group and the group without VCF (*P*=0.66, P=0.35 and *P*= 0.21, respectively). When assessing the differences in rates of change, there was a significant change in slope for PDFF (2.32 per 6 months, 95% confidence interval (CI) 0.31-4.32; P=0.03) but not for T2* (0.02 per 6 months, CI -0.98-0.95; P=0.90) or BMD (-4.84 per 6 months, CI -23.4-13.7; P=0.60).

**Conclusions:**

In our study population, the average change of PDFF over 12 months is significantly higher in patients that develop incidental fractures at 12-month follow up compared to patients without incidental VCF, while T2* and BMD show no significant changes prior to the occurrence of the incidental vertebral fractures. Therefore, a longitudinal increase in bone marrow PDFF may be predictive for vertebral compression fractures.

## Introduction

In our rapidly aging population, vertebral compression fractures (VCFs) are increasing in frequency due to reduced bone strength as a result of osteoporosis (1). Osteoporosis is a systemic disease that leads to reduced bone mass and microarchitectural deterioration of bone tissue and as a result, low-energy (or even no evident) trauma can lead to VCFs, which affects up to 25% of all postmenopausal women (2–4). Assessment of the bone is of high importance, since reduced bone strength can be treated, reducing the risk for fractures and following complications such as pain and immobilisation (5), being a burden for the individual patient as well as for the health care and social security systems (6, 7).

In the clinical routine, quantitative assessment of the bone structure predominantly relies on dual-energy X-ray absorptiometry (DXA) and dedicated quantitative CT (QCT), which are the clinical standards for determining the bone mineral density (BMD) (6). However, quantitative MR techniques have been introduced and investigated for analysis of vertebral bone marrow, such as chemical shift encoding-based water–fat MRI (CSE-MRI). CSE-MRI is a non-invasive approach to extract precise quantitative information about water–fat compositions of the vertebral bone marrow by determining the proton density fat fraction (PDFF), showing very good concordance with histology or magnetic resonance spectroscopy (8–12). CSE-MRI therefore enables *in vivo* quantitative evaluation of the bone marrow without exposing the patient to ionizing radiation. PDFF measurements of the vertebral bone marrow have proved to be particularly interesting in the analysis of osteoporosis, as the composition of the vertebral bone marrow, consisting of a mixture of hematopoietic tissue and adipocytes interspersed within the trabecular bone matrix, shifts towards a higher fat content (12, 13). Previous studies demonstrate that PDFF derived by CSE-MRI represents a potential biomarker for quantification of osteoporosis, bone fragility, and assessment of osteoporotic fractures (14–16), without exposing the patient to radiation when compared to DXA and QCT. Those techniques also enable the assessment of bone marrow T2* (3, 17–19), which has been shown to correlate with the microarchitectural structure of the vertebral body (20).

However, all previous studies were cross-sectional studies that only included patients without fractures or patients already suffering from VCFs. To the best of our knowledge, there is no study that has analyzed the intraindividual dynamic of the chemical composition in the vertebral bone marrow using chemical shift encoding-based water-fat separation techniques yet, especially prior to the occurrence of a VCF. It would be of great interest, whether this technique allows for prognostic assessment in regard to risk evaluation for future VCFs. We hypothesize that there might be measurable changes in PDFF or T2* in the vertebral bone marrow prior to the occurrence of a VCF, which, as a result, could identify patients at risk and prevent imminent VCFs.

Therefore, the purpose of this study was to investigate the association between incidental VCFs and longitudinal changes in PDFF and T2* measurements prior to the occurrence of vertebral fractures in the thoracolumbar spine.

## Methods

### Patient selection and study design

**Figure 1:**
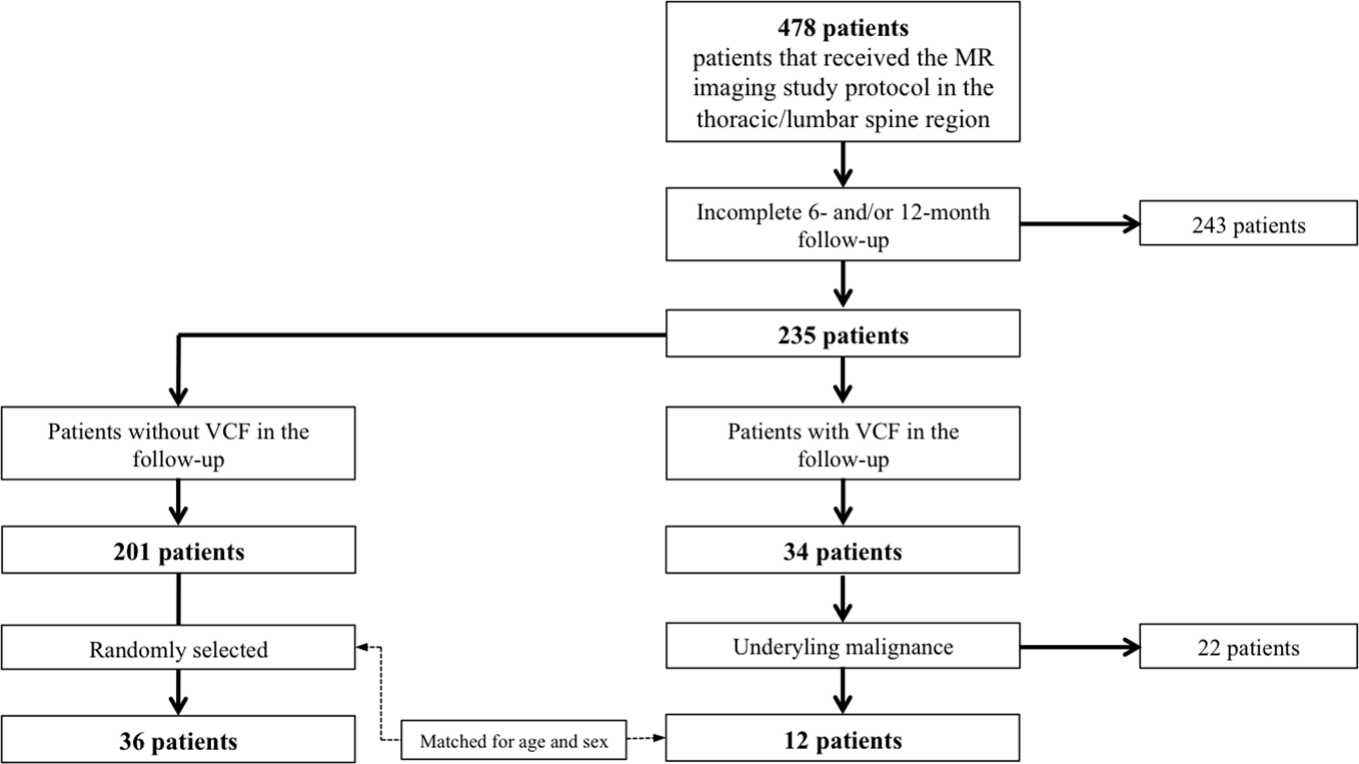
Flowchart of patient selection.

In our institution, from June 2018 to December 2021, a total number of 478 patients received an abdominal MR imaging protocol including a multi-echo gradient-echo sequence of the thoracolumbar spine, as described below. The inclusion criteria were: i) occurrence of an incidental VCF that was ii) not present in the 2 MRI examinations prior to the VCF and iii) without evidence for a high-energy traumatic or malignant etiology (i.e. osseous metastasis or primary bone tumor). Ultimately, 12 patients (70.5 ± 7.4 years, 5 females) with incidental vertebral fractures were identified that met all the criteria (**Figure 1**). Those patients were frequency-matched (1:3) for age and sex to patients without incidental VCFs (n=36, 71.1 ± 8.6 years, 15 females). History of medical treatment was checked in all included patients. None of the patients had received chemotherapy prior to or during the study. All patients underwent 3T-MRI of the abdomen including axially acquired gradient echo sequences for chemical shift encoding-based water-fat separation, from which PDFF and T2* maps of the thoracolumbar spine were obtained. The first imaging session was defined as the baseline MR imaging and follow-up sessions were performed every 6 months (6.7 ± 3.4 months). The study was approved by the local institutional review board (Ethics Commission of the Medical Faculty, Technical University of Munich, Germany; Ethics proposal number 2022-433-S-SR).

### Magnetic resonance imaging and T2*/PDFF assessment

Two 3T-MRI systems (one Ingenia, Philips Healthcare and one Elition, Philips Healthcare) were used for the examination of the abdomen including the lumbar spine. The patients were placed in supine position and the combination of a 16-channel torso coil array and an inbuilt table posterior 12-channel coil array was used. The imaging sessions were scheduled according to the clinical follow-up, which includes CSE-MRI for PDFF and T2* measurement. For the PDFF and T2* measurements a six-echo 3D multi-echo gradient-echo sequence was used acquiring all echoes in a single TR using bipolar gradients with the following parameters: repetition time TR/first echo time TE1/echo time step ΔTE = 7.8/1.35/1.1ms, field of view (FOV)=300 x 400 x 150 mm^3^, acquisition voxel size = 2 x 3 x 6 mm^3^, reconstruction voxel size = 1.13 x 1.13 x 6 mm^3^, receiver bandwidth = 1678 Hz/pixel, frequency direction = anterior/posterior (A/P), 1 average, scan time = 9.3 s. To minimize T1-bias effects a flip angle of 3° was used (21).

Complex multi-echo gradient-echo images were provided as input to the fat quantification routine provided by the vendor (mDixon Quant, Philips Healthcare). Specifically, after phase correction, a complex-based water-fat decomposition was performed using a single T2* correction and a pre-calibrated fat spectrum accounting for the presence of the multiple peaks in the fat spectrum. A seven-peak fat spectrum model was employed (22). The PDFF map was computed as the ratio of the fat signal over the sum of fat and water signals. PDFF and T2* maps were extracted.

Furthermore, the MR imaging protocol encompasses an axial and coronal T2-TSE sequence as well as axially acquired native and contrast-enhanced T1 weighted sequences with spectral fat saturation according to clinical standards.

**Figure 2:**
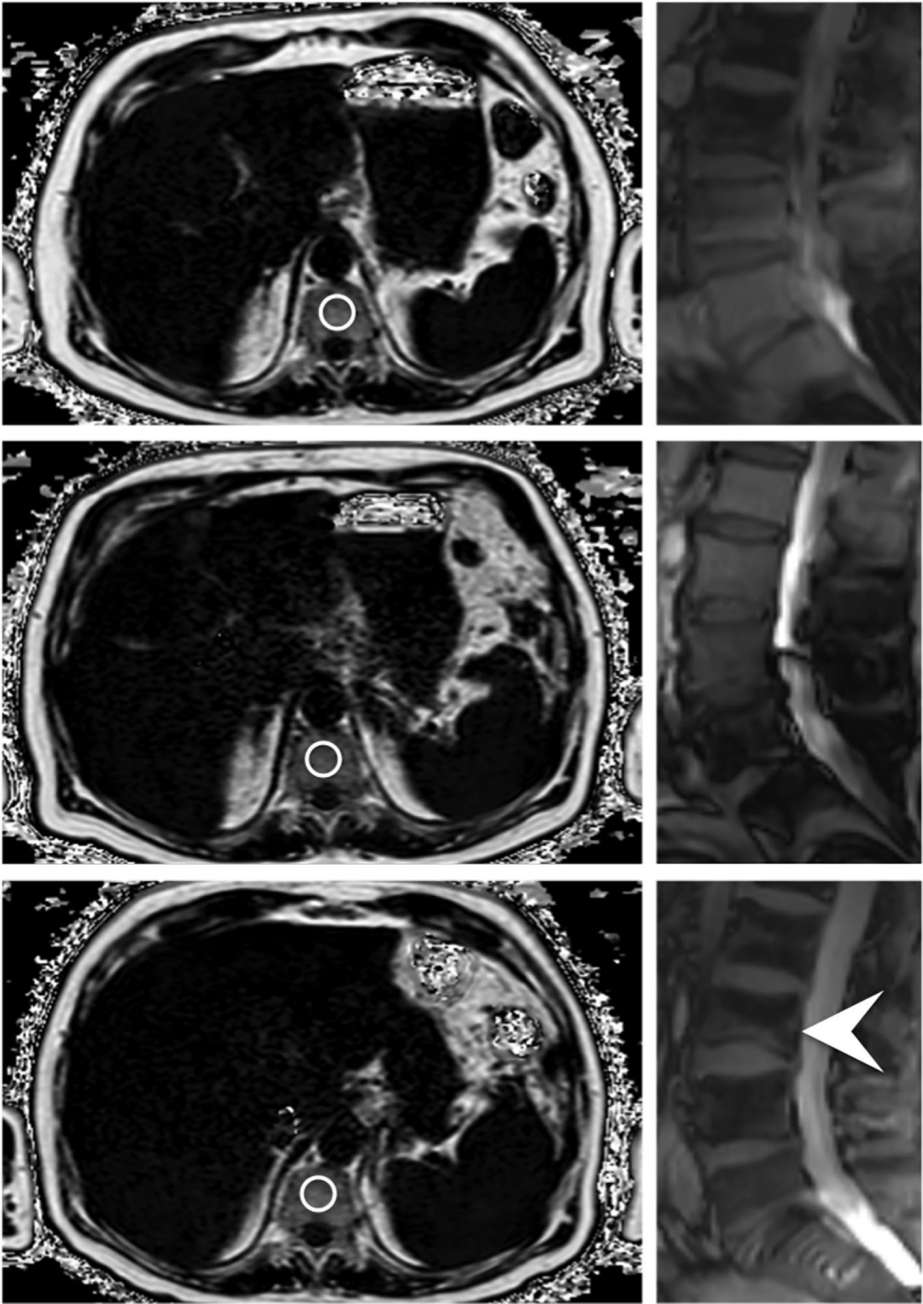
Axially acquired gradient echo sequences for chemical shift encoding-based water-fat separation of a 78 year old male patient at baseline, first follow-up and at the time of fracture occurrence; the images on the right originate from the corresponding survey in sagittal orientation, which reveal an incidental VCF of the base plate of L3 (indicated by the white arrowhead). The circles centrally placed in the vertebrae represent ROIs used for the PDFF measurements.

Segmentations were performed on axial reformations (**Figure 2**). The vertebral bodies Th11 to L4 were manually segmented by F.T.G. and Y.L. (3 and 4 years of experience in musculoskeletal imaging). For this, cylindrical ROIs were placed in the center of each vertebra and the mean PDFF and T2* values were noted for each vertebra. The measurements from Th11 to L4 were then averaged for each examination. Fractured or otherwise altered vertebrae (e.g. after kyphoplasty, heavy degenerative alterations; n=18) were excluded.

The morphologic imaging features of the vertebral fractures (involvement of the superior and/or inferior endplate; deformity (e.g. wedge, biconcave); involvement of the posterior column; Genant classification (mild, moderate, severe)) were assessed by two board-certified radiologists (B.J.S. and A.S.G., with 11 years and 10 years of experience in musculoskeletal imaging). The radiologists ensured, in context with the clinical history, that no morphologic signs for a malignant fracture were present in any of the sequences.

In order to assess the intra- and inter-reader reproducibility error of the PDFF, T2* and BMD values, a random sample of 10 subjects was chosen and reanalyzed by the same radiologist.

### QCT and BMD measurements

BMD measurements were performed in patients that received CT in a time period ± 1 month around the MR imaging sessions. CT images were acquired with one dual-layer dual-energy CT scanner (IQon Spectral CT, Philips Healthcare) and one multislice detector CT scanner (iCT 256, Philips Healthcare). CT was performed according to our routine clinical protocols: collimation, 0.6 mm; pixel spacing, 0.4/0.3 mm; pitch factor, 0.8/0.9; tube voltage (peak), 120 kV; modulated tube current, 102–132 mA. Images were reformatted in 3-mm slice thickness using a bone-specific convolution kernel.

For BMD measurements, a mid-line 15mm MPR section in sagittal reformations was created with a PACS tool (IDS7, Sectra). Cylindrical volumes of interest were manually positioned in the non-fractured lumbar vertebrae by one radiologist (G.C.F.), and mean Hounsfield Units (HU) were noted (23). Fractured or otherwise altered vertebrae (e.g. vertebrae with severe degenerative changes, vertebrae after vertebro-/kyphoplasty) were excluded. The HU values were then converted into BMD using conversion equations as previously described: (i) 0.928 x HU + 4.5 mg/cm^3^ for the IQon Spectral CT, (ii) 0.855 x HU + 1.172 mg/cm^3^ for the Philips iCT 256 (24, 25). Osteoporosis was defined as BMD < 80 mg/cm^3^ and osteopenia as 80 ≤ BMD ≤ 120 mg/cm^3^ (26).

### Statistical analysis

Statistical analysis was performed with SPSS version 28 software (IBM) using a two-sided 0.05 level of significance. One-way analysis of variance (for parametric variables) and chi-square tests (for categorical variables) were used to evaluate differences in patient characteristics between patients with and without incidental fractures. Associations between changes in PDFF, T2* and BMD measurements over 12 months and the group (incidental VCF vs. no incidental VCF) were assessed using multivariable regression models adjusting for age and sex. Moreover in order to assess the validity of our hypothesis, we used mixed-effect regression models with random intercepts to test if there is a difference in the rate of change in PDFF, T2* and BMD between patients with and without incidental VCF.

Intrarater and interrater reproducibility for T2*, PDFF and trabecular BMD values were assessed by calculating the intraclass correlation coefficient and the root mean square coefficient of variation (RMSCV) of the differences between the respective measurements.

## Results

In this study, 12 patients (70.5 ± 7.4 years, 5 female) with incidental VCFs were identified and were frequency-matched (1:3) for age and sex to subjects without vertebral fractures (n=36, 71.1 ± 8.6 years, 15 females) (**Table 1**). Of all incidental VCFs, 16.7% were located in Th12, 25% in L1, 16.7% in L2, 33.3% in L3 and 8.3% in L4.

**Table 1:** Patient characteristics.

Characteristics	with incidental VCF (n = 12)	without incidental VCF (n=36)	all (n = 48)
Sex female male	5 7	15 21	20 28
Age (years)	70.5 ± 7.4	71.1 ± 8.6	70.9 ± 8.2

### PDFF and T2* measurements

**Figure 3:**
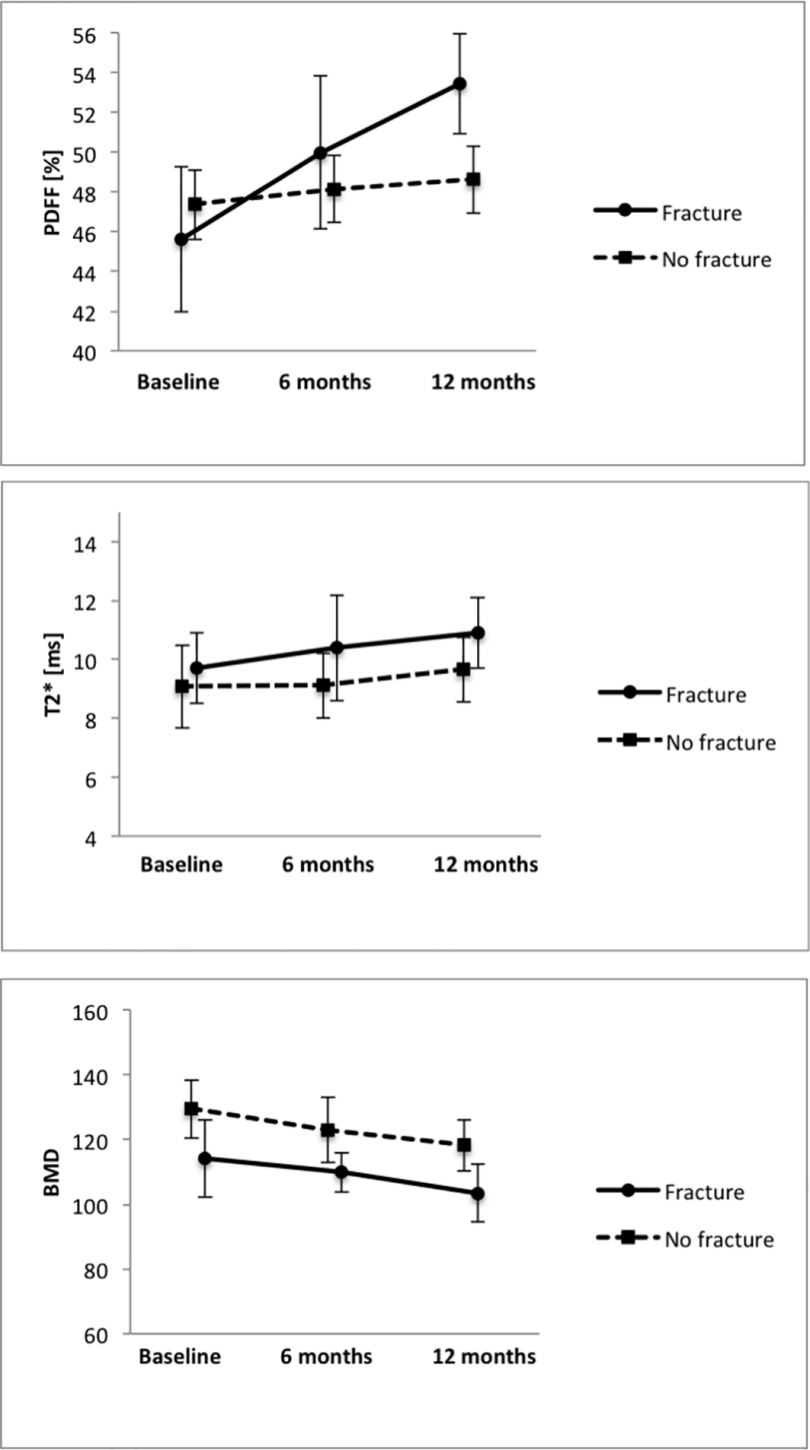
Average PDFF, T2* and BMD measurements in the groups with and without VCFs at baseline, 6-month and 12-month follow-up.

No significant difference was detected in the average PDFF of all measured vertebrae (Th11-L4) between the group with incidental VCFs and the group without VCF at baseline, 6-month and 12-month follow-up (baseline, PDFF=45.6 ± 12.6% in the VCF group vs. PDFF=47.4 ± 10.5% in the no-VCF group; 6-month follow up, PDFF=50.0 ± 13.3% vs. PDFF=48.1 ± 10.1%; 12-month follow-up, PDFF=53.4 ± 8.7% vs. PDFF=48.6 ± 10.1%; *P*=0.66, *P*=0.65 and *P*=0.24 respectively) (**Table 2**). Moreover, there was no significant difference in T2* measurements (baseline, T2*=9.7 ± 2.1ms vs. T2*=9.1 ± 2.0ms; 6-month follow-up, T2*=10.4 ± 3.0ms vs. T2*=9.1 ± 1.8ms; 12-month follow-up T2*=10.9 ± 1.5ms vs. T2*=9.7 ± 1.8ms; *P*=0.35, *P*=0.10 and *P*=0.58 respectively) (**Figure 3**).

**Table 2:** PDFF, T2* and BMD values at baseline, 6-month follow-up and 12-month follow-up/time of fracture.

Measured value	with incidental VCF	without incidental VCF	p-value
PDFF (%) baseline 6-month follow-up 12-month follow-up	45.6 ± 12.6 50.0 ± 13.3 53.4 ± 8.7	47.4 ± 10.5 48.1 ± 10.1 48.6 ± 10.1	0.66 0.65 0.24
Δ_PDFF_	6.3 ± 3.1	2.1 ± 2.5	0.03*
T2* (ms) baseline 6-month follow-up 12-month follow-up	9.7 ± 2.1 10.4 ± 3.0 10.9 ± 1.5	9.1 ± 2.0 9.1 ± 1.8 9.7 ± 1.8	0.35 0.10 0.58
Δ_T2*_	1.7 ± 1.1	1.1 ± 1.3	0.31
BMD (mg/cm^3^) baseline 6-month follow-up 12-month follow-up	114.1 ± 28.8 109.8 ± 8.4 103.4 ± 25.5	129.5 ± 22.8 122.9 ± 15.8 118.2 ± 30.1	0.21 0.43 0.34
Δ_BMD_	-1.2 ± 11.3	-11.4 ± 24.1	0.37

Δ-values describe the average of the change of PDFF, T2* and BMD between baseline and 12-month follow-up for every patient.

**Figure 4:**
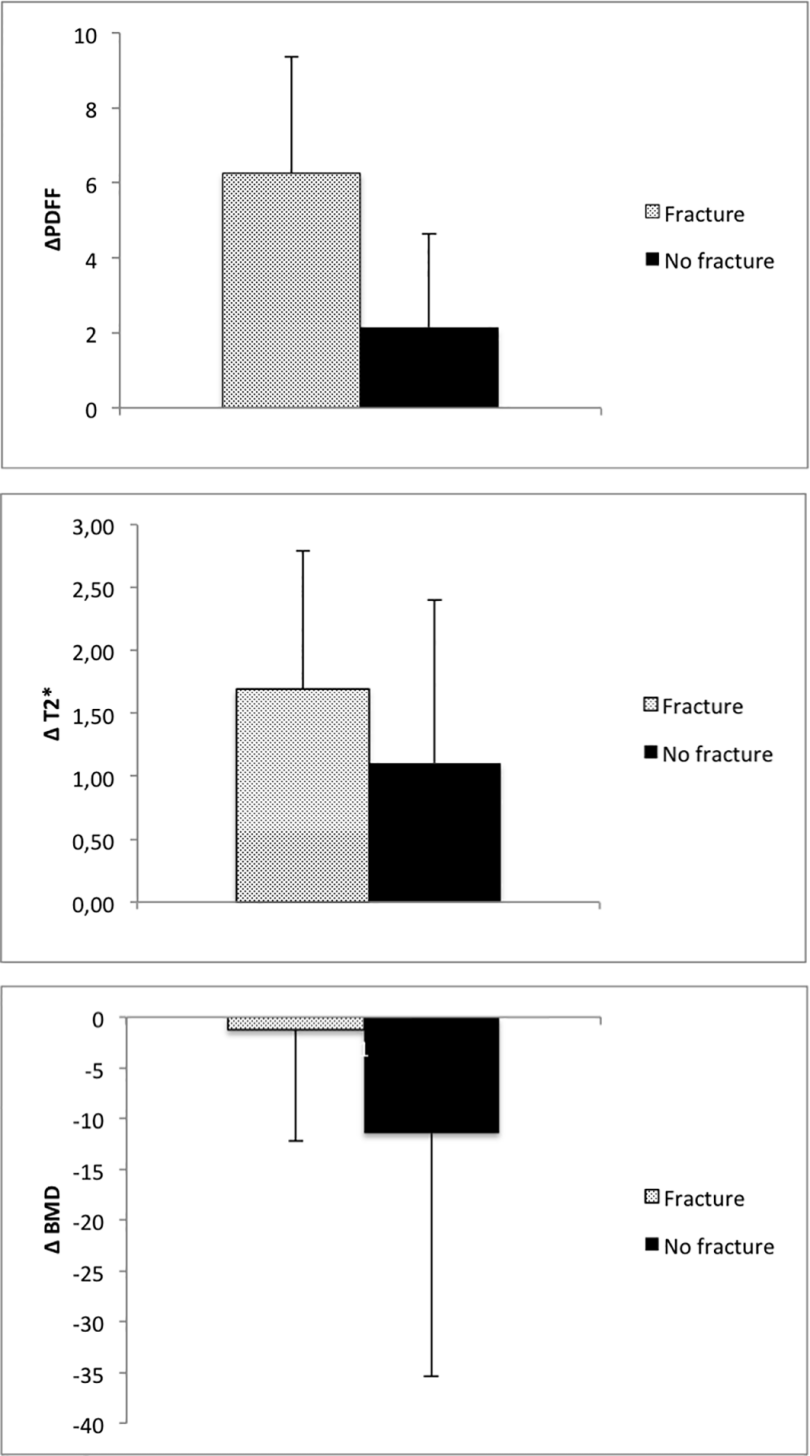
Average change of PDFF, T2* and BMD in patients with and without incidental VCF between baseline and 12-month follow-up.

When analyzing the change of PDFF between the baseline MR examination and the 12-month follow-up, there was an average increase in vertebral PDFF of Δ_PDFF_=6.3 ± 3.1% in the group with incidental VCFs, which is significantly higher (*P*=0.03) than the change in PDFF in the group without VCFs (2.1 ± 2.5%, **Figure 4**; **Table 2**). However, there was no significant difference regarding the changes of vertebral T2* between the group with and without incidental VCF at follow up (Δ_T2*_=1.7 ± 1.1ms vs. Δ_T2*_=1.1 ± 1.3ms, *P*=0.31). When assessing the differences in rates of change of the PDFF and T2* values over 12 months between patients with and without incidental VCFs, there was a significant change in slope for PDFF (2.32 per 6 months, 95% confidence interval (CI) 0.31-4.32; P=0.03) but not for T2* (0.02 per 6 months, CI -0.98-0.95; P=0.90).

### BMD measurements

BMD of the spine was measured using opportunistic QCT, if CT imaging was performed in proximity to the MR examinations. There was no significant difference between the two groups regarding the BMD measurements at baseline, 6-month or 12-month follow-up (baseline, 114.1 ± 28.8mg/cm^3^ vs. 129.5 ± 22.8mg/cm^3^; 6-month follow-up, 109.8 ± 8.4mg/cm^3^ vs. 122.9 ± 15.8mg/cm^3^; 12-month follow-up, 103.4 ± 25.5mg/cm^3^ vs. 118.2 ± 30.1mg/cm^3^; *P*=0.21, P=0.43 and P=0.34 respectively). Moreover, when assessing the differences in BMD change over 12 months, there was no significant difference between the group with and without incidental VCF (-1.2 ± 11.3 mg/cm^3^ vs. -11.4 ± 24.1 mg/cm^3^, *P*=0.37). When assessing the differences in rates of change of the BMD values over 12 months between patients with and without incidental VCFs, there was no significant change in slope (-4.84 per 6 months, CI -23.4-13.7; P=0.60).

Also, according to the above-described data, there was no indication for a higher prevalence of osteoporosis in either group. At baseline, one of the patients with an incidental VCF (8.3%) showed an osteoporotic BMD (<80mg/cm^3^), whereas 3 patients in the group without fracture had BMD values that qualified for osteoporosis.

### Intrareader reproducibility

The intrareader (ICC, 0.99 [95% CI, 0.98–0.99]) and interreader (ICC, 0.99 [95% CI, 0.97–0.99]) agreement for trabecular BMD as well as the intrareader and interreader agreement for PDFF (ICC for both, 0.98 [95% CI, 0.96–0.99]) and T2* measurements (ICC for both, 0.98 [95% CI, 0.96–0.99]) were excellent. Intrarater reproducibility, calculated with the RMSCV, was 0.4% for the BMD analysis and 0.9% for the mean PDFF analysis and 0.8% for the mean T2* analysis. The interreader agreement for PDFF, calculated with RMSCV, was 0.8%, the interreader agreement for T2* was 0.9% and the respective interreader agreement for the trabecular BMD was 0.5%.

## Discussion

In this study, we investigated longitudinal changes over 12 months of PDFF and T2* measurements using chemical shift encoding-based water-fat separation for the assessment of patients with and without incidental vertebral compression fractures. Our study demonstrates that the average change of PDFF over 12 months is significantly higher in patients that develop incidental fractures at 12-month follow up compared to patients without incidental VCF, while T2* and BMD show no significant changes prior to the occurrence of the incidental vertebral fractures. Our data suggests that PDFF, when evaluated longitudinally in the same patient, might indicate changes in the vertebral structural integrity and composition very early and therefore might be a sensitive biomarker for the identification of patients at risk for incidental VCFs.

Past studies have shown that vertebral bone marrow PDFF might be a useful parameter for assessment of osteoporosis and reduced bone strength (14–16). PDFF derived from chemical shift encoding-based water-fat separation measures a map of the density of hydrogen protons attributable to fat, normalized by the total hydrogen proton density from all mobile proton species (27). PDFF enables a fairly precise estimation of the fat volume fraction as a result of the almost equal relative proton densities of fat and water (8). Therefore, it has been proposed to be a non-invasive tool for assessment of e.g. hepatic steatosis and, as previous studies demonstrate, osteoporosis or low-energy fractures (13, 18, 28, 29).

In general, osteoporosis is characterized by a reduced bone mass and microarchitectural deterioration of bone tissue. The lost bone mass in the vertebral space is not just filled with fatty bone marrow but rather includes mechanisms such as a drift in mesenchymal stem cell differentiation towards adipogenesis over osteoblastogenesis (30, 31). Multiple studies have shown that there is a negative correlation between PDFF values and BMD in vertebral bone marrow; as a result this technique enables a differentiation of patients with osteoporosis and healthy subjects (32). Another study demonstrated that PDFF allows the differentiation between osteoporotic patients with and without vertebral fractures, representing a potentially useful radiation-free tool for the assessment of bone fragility in addition to BMD measurements (16). CSE-MRI also enables T2* measurements, which have shown to be indirect measures of vertebral bone architecture, as the local magnetic field inhomogeneities induced by the trabecular bone and bone marrow interface can be measured as a shortening of the effective transverse relaxation time. T2* measurements correlated with the bone mineral density in previous studies, where T2* increases with decreasing bone density (19). However, all previous studies were cross-sectional studies that only included patients without fractures or patients already suffering from VCFs. To the best of our knowledge, there is no study that has analyzed the intraindividual dynamic of the composition of vertebral bone marrow using chemical shift encoding-based water-fat separation techniques in MR imaging yet, especially prior to the occurrence of a VCF.

In past studies opportunistic QCT was evaluated for patients at risk for recurring osteoporotic VCFs (24, 33, 34). In our patient cohort however, BMD measured by QCT and T2* were not able to predict occurrence of incidental fractures longitudinally. Also, according to our data, patients with incidental fractures do not differ from subjects without VCFs in regard to the overall average PDFF. A tendency is observable in the fracture group with a rise in average PDFF, even though it is not statistically different from the non-fracture group. Only the relative change of PDFF showed a significant difference between patients with imminent fractures compared to patients without fractures. We therefore hypothesize that PDFF may be a very sensitive biomarker that may be useful for the prediction of the occurrence of incidental fractures. Yet larger longitudinal study cohorts are needed in order to investigate these results further.

One important limitation of our study is the relatively small number of patients with VCFs, although being a frequent condition especially in older patients. However, this problem is difficult to eliminate since the occurrence of a VCF in a patient population that is not explicitly at risk for VCFs beforehand is still a stochastic event; a very large number of patients is necessary that repeatedly receives MR examinations until a sufficiently large patient number with VCFs is gathered. Another limitation is the incomplete data set for BMD measurements using QCT; as the patients already received MR examinations there was often no justifying indication for CT, which would lead to unnecessary radiation exposure. It is therefore necessary to confirm our findings in future studies with a larger data set.

In conclusion, in our study population the average change of PDFF was significantly higher in patients that developed incidental fractures compared to patients without incidental VCFs, while T2* and BMD showed no significant changes. Therefore, when evaluated longitudinally, PDFF might indicate changes in the vertebral structural integrity and composition very early and might be a sensitive biomarker for the identification of patients at risk for incidental VCFs. Yet, this needs to be further assessed in larger longitudinal study groups.

## Data availability statement

The raw data supporting the conclusions of this article will be made available by the authors, without undue reservation.

## Ethics statement

The studies involving human participants were reviewed and approved by Ethics Commission of the Medical Faculty, Technical University of Munich, Germany (Ethics proposal number 2022-433-S-SR). Written informed consent for participation was not required for this study in accordance with the national legislation and the institutional requirements.

## Author contributions

Conceptualization, AG, YL; methodology, AG, YL, TB, JSK, DK; validation, TB, JSK, FTG, BS, SR, DK; formal analysis, YL, JK, LG, FTG, FGG, GF; investigation, YL, JK, LG, FTG, FGG, GF; resources, AG, RB, DK, MM; data curation, YL, JK, LG, FTG, FGG, GF; writing—original draft preparation, YL; writing—review and editing, all authors; visualization, YL, JK; supervision, AG; project administration, YL, AG; funding acquisition, AG, MM. All authors contributed to the article and approved the submitted version.

## Conflict of interest

JSK is Co-Founder of BoneScreen GmbH.

The remaining authors declare that the research was conducted in the absence of any commercial or financial relationships that could be construed as a potential conflict of interest.

## Publisher’s note

All claims expressed in this article are solely those of the authors and do not necessarily represent those of their affiliated organizations, or those of the publisher, the editors and the reviewers. Any product that may be evaluated in this article, or claim that may be made by its manufacturer, is not guaranteed or endorsed by the publisher.
